# Detection of BK polyomavirus-associated nephropathy using plasma graft-derived cell-free DNA: Development of a novel algorithm from programmed monitoring

**DOI:** 10.3389/fimmu.2022.1006970

**Published:** 2022-10-06

**Authors:** Jingyu Wen, Rongcun Sun, Hongji Yang, Qing Ran, Yifu Hou

**Affiliations:** ^1^ Department of Medical Insurance, Sichuan Academy of Medical Sciences and Sichuan Provincial People’s Hospital, University of Electronic Science and Technology of China, Chengdu, China; ^2^ School of Medicine, University of Electronic Science and Technology of China, Chengdu, China; ^3^ Department of Organ Transplantation, Sichuan Academy of Medical Sciences and Sichuan Provincial People’s Hospital, University of Electronic Science and Technology of China, Chengdu, China; ^4^ Clinical Immunology Translational Medicine Key Laboratory of Sichuan Province & Organ Transplantation Center, Sichuan Academy of Medical Sciences and Sichuan Provincial People’s Hospital, Chengdu, China

**Keywords:** graft-derived cell-free DNA, BK polyomavirus-associated nephropathy, programmed monitoring, algorithm, diagnosis

## Abstract

Graft-derived cell-free DNA (GcfDNA) is a promising non-invasive biomarker for detecting allograft injury. In this study, we aimed to evaluate the efficacy of programmed monitoring of GcfDNA for identifying BK polyomavirus-associated nephropathy (BKPyVAN) in kidney transplant recipients. We recruited 158 kidney transplant recipients between November 2020 and December 2021. Plasma GcfDNA was collected on the tenth day, first month, third month, and sixth month for programmed monitoring and one day before biopsy. ΔGcfDNA (cp/mL) was obtained by subtracting the baseline GcfDNA (cp/mL) from GcfDNA (cp/mL) of the latest programmed monitoring before biopsy. The receiver operating characteristic curve showed the diagnostic performance of GcfDNA (cp/mL) at biopsy time and an optimal area under the curve (AUC) of 0.68 in distinguishing pathologically proven BKPyVAN from pathologically unconfirmed BKPyVAN. In contrast, ΔGcfDNA (cp/mL) had a sensitivity and specificity of 80% and 84.6%, respectively, and an AUC of 0.83. When distinguishing clinically diagnosed BKPyVAN from clinical excluded BKPyVAN, the AUC of GcfDNA (cp/mL) was 0.59 at biopsy time, and ΔGcfDNA (cp/mL) had a sensitivity and specificity of 81.0% and 76.5%, respectively, and an AUC of 0.81. Plasma ΔGcfDNA (cp/mL) was not significantly different between TCMR [0.15 (0.08, 0.24) cp/mL] and pathologically proven BKPyVAN[0.34 (0.20, 0.49) cp/mL]. In conclusion, we recommend programmed monitoring of plasma GcfDNA levels after a kidney transplant. Based on our findings from the programmed monitoring, we have developed a novel algorithm that shows promising results in identifying and predicting BKPyVAN.

## Introduction

BK polyomavirus (BKPyV) infection frequently causes BK polyomavirus-associated nephropathy (BKPyVAN) in up to 10% of kidney transplant recipients (KTRs) (1). BKPyV is a significant risk factor for kidney allograft dysfunction and loss among KTRs (2). The primary method for detecting BKPyV infection is quantitative polymerase chain reaction (PCR) to detect BKPyV DNA in urine and plasma. However, the PCR results cannot be used for diagnosing BKPyVAN. The diagnosis of BKPyVAN relies on the histopathological detection of simian virus 40 (SV40) in kidney biopsy tissues. However, this has several disadvantages, including invasive procedures, high false-negative rates, and sampling errors. In other words, a negative anti-SV40 stain does not exclude BKPyVAN, especially when infected renal tubules cannot be obtained or no significant infection has been formed in the renal cortex, which will affect the accuracy of pathology. Therefore, some cases cannot be diagnosed by pathological biopsy. At the same time, some kidney transplant centers do not perform kidney graft biopsies as part of their routine follow-up procedures. Therefore, non-invasive biomarkers are urgently needed to assist in the diagnosis of BKPyVAN.

Graft-derived cell-free DNA (GcfDNA) originates from allografts and is considered a potential noninvasive marker for evaluating graft injury, especially in distinguishing antibody-mediated rejection (ABMR) (3–10). As with graft rejection, graft injury caused by BKPyV may also lead to increased GcfDNA levels in the urine or blood. Apart from BKPyV, there is little research on the relationship of GcfDNA levels with other viruses such as Cytomegalovirus or Epstein–Barr virus after kidney transplantation (11). Chen et al. reported that urine GcfDNA concentration in BKPyVAN was higher than that in T cell-mediated rejection (TCMR), borderline change, and negative biopsies (12). Chen et al. reported that elevated urine GcfDNA levels may help to distinguish BKPyVAN in kidney transplant recipients with BKPyV viruria (13). Kant et al. suggested that GcfDNA may be a useful noninvasive test to assess progression of BKPyV to BKPyVAN (14). BKPyVAN can induce moderate increases in GcfDNA (15). Goussous et al. suggested that elevations in GcfDNA are not specific to kidney allograft rejection and can be associated with BK viremia affecting the transplanted kidney (16). However, these studies mainly focused on urine GcfDNA, which is associated with poor reproducibility and a low DNA extraction rate, and were primarily assessed on a “for-cause” basis but not a “for-protocol” basis. More importantly, GcfDNA tends to rise before clinical symptoms, so it may not be appropriate to assess GcfDNA when BKPyV-related kidney injury is suspected.

Thus far, the role of plasma GcfDNA in the diagnosis of BKPyVAN has not been confirmed. We performed this study to investigate the value of plasma GcfDNA level in programmed monitoring and during BKPyV infection. The diagnostic capabilities of plasma GcfDNA in BKPyVAN were further analyzed and used to develop algorithms to detect BKPyVAN.

## Materials and methods

### Study design and patients

We enrolled primary KTRs at the organ transplant center of Sichuan Provincial People’s Hospital (Chengdu, China). The inclusion criteria were as follows: (a) adult male or female aged > 18 years and (b) voluntary participation with informed consent. The exclusion criteria were as follows: (a) multi-organ transplant, (b) repeated kidney transplant, (c) severe pneumonia, (d) refusal to participate in the study, and (e) loss to follow-up. Urine BKPyV infection in our study included patients with urine BKPyV load > 3 log_10_ copies/mL. Those with urine BKPyV DNA load > 7 log_10_ copies/mL would undergo further kidney biopsy to confirm the diagnosis of BKPyVAN (biopsy cohort). Written informed consent was obtained from all the patients. Kidney allografts from living or deceased organ donors who met the ethical guidelines for kidney donation were used in this study. No kidney transplant recipients received donor organs from executed prisoners or other institutionalized individuals. This study adhered to the tenets of the Declaration of Helsinki and was approved by the Ethics Committee of the Sichuan Provincial People’s Hospital (No. 2020405).

### Sample collection and DNA extraction

For each recipient, 10 mL peripheral blood samples were collected regularly on the tenth day (D10), first month (M1), third month (M3), and sixth month (M6) after transplantation. A kidney biopsy was required for KTRs who were diagnosed with a urine BKPyV DNA load > 7 log_10_ copies/mL during the postoperative follow-up. Additional blood samples for GcfDNA were collected 1 d before kidney biopsy (biopsy time). Within 10 h of blood collection, plasma was separated by centrifugation for 10 min at 2000 × *g*, after which the supernatant was centrifuged for 20 min at 4000 × *g*. Then, the plasma was stored at −80°C until circulating cell-free DNA (cfDNA) was extracted. cfDNA was extracted using the QIAamp Circulating Nucleic Acid Kit (QIAGEN, Germany), according to the manufacturer’s instructions.

### Quantification of GcfDNA

The fractional abundance (%) and genomic copies per mL plasma (cp/mL) of the GcfDNA were measured using a technical testing service called “YiLeShu-Graft sentinal^®^” by S&KM Biotechnology Co., Ltd. GcfDNA fractions (%) were calculated using the following formula:


$$GcfDNA\;fractions(\%)\;=\frac{Donor\;cfDNA\;(copy\;number\;of\;homozygous\;genotype\;representing\;graft-derived\;cfDNA\;in\;selected\;SNP)}{Total\;cfDNA\;(total\;copy\;number\;of\;selected\;SNP)}$$

Absolute quantification of haploid GcDNA genomic copies per mL plasma (cp/mL) is calculated by multiplying the total concentration of cfDNA (cp/mL) in a sample by the GcfDNA fraction (%). The concentration of the total cfDNA is determined by a digital droplet PCR. The assay was performed using the Bio-Rad QX200 Droplet Digital System (Bio-Rad Laboratories, US), and the data were processed using QuantaSoft™ version 1.7.4 software (Bio-Rad Laboratories, US).

### Diagnosis of BKPyVAN

The pathological diagnosis of BKPyVAN is according to the 2019 American Society of Transplantation guidelines in (**Table 1**) (17). Proven BKPyVAN will also be referred to as pathologically proven BKPyVAN, and the other categories will be considered as pathologically unconfirmed BKPyVAN. Pathological lesions were scored according to the 2017 Banff criteria (18). The pathological features of BKPyVAN were classified using the American Society for Transplantation schema; BKPyVAN was classified as stage A, B, or C, based on the Banff guidelines published by Hirsch et al. (19).

**Table 1:** BKPyVAN diagnostic guideline.

Pathological diagnose	Urine BKPyV DNA load	Plasma BKPyV DNA load	Anti-SV40 IHC stain
possible BKPyVAN	> 7 log10 copies/mL	negative	negative
probable BKPyVAN	> 7 log10 copies/mL	> 3 log10 copies/mL in two measurements wiSthin 3 weeks	negative
presumptive BKPyVAN	> 7 log10 copies/mL	> 4 log10 copies/mL in at least one of two measurements in< 3 weeks	negative
proven BKPyVAN	> 7 log10 copies/mL	> 4 log10 copies/mL in at least one of two measurements in< 3 weeks	positive

BKPyVAN, BK polymavirus-associated nephropathy;BKPyV, BK polymavirus; IHC, immunohistochemistry.

The detection method of IHC staining is as follows: 2–3 µm paraffin sections, dewaxed in water, were thermally repaired with EDTA antigen repair solution (pH 8.0) for 3–5 min. An appropriate amount of endogenous peroxidase blocker was added and incubated at room temperature for 10 min, after which an appropriate amount of primary antibody SV40 was added and incubated overnight at 4°C. Next, an appropriate amount of secondary antibody was added and incubated at 37°C for 30 min; DAB chromogenic solution was added to the sections and incubated for 5 min at room temperature; they were then counterstained with hematoxylin and sealed.

We used the BK virus nucleic acid detection kit and real-time fluorescent PCR probe (TaqMan) technology to detect BK virus DNA quantitatively. For the plasma sample, 35 mL of the patient’s venous blood was drawn, and the detection was completed within 4 h at room temperature. For urine samples, we obtained 10–20 mL of interrupted morning urine and completed the detection within 24 h at room temperature. The minimum detection limit of the kit is 2 × 10^3^ copies/mL, and the upper limit is 5 × 10^8^ copies/mL.

Considering the fact that the pathological diagnosis of BKPyVAN was far from perfect, patients with negative pathological results who responded to CNI dose reduction could be diagnosed with clinical BKPyVAN if drug-induced renal injury and other possible causes were ruled out.

### Statistical analysis

Continuous variables with normal distributions were presented as means with standard deviation (SD) and evaluated using an independent *t*-test. Skewed distributed variables were presented as medians (interquartile range [IQR]) and analyzed using the Mann–Whitney *U* test. Categorical variables were presented as percentages and were tested using the χ^2^ test. The Kolmogorov–Smirnov normality test was used to assess for normality. Pearson’s test was used to identify correlations among variables. Logistic regression modeling was used for binary covariates. Discrimination was quantified using the area under the curve (AUC) of the receiver operating characteristic (ROC) curve. The least absolute shrinkage and selection operator (LASSO) regression was used to predict BKPyVAN after kidney transplantation using various clinical variables and early GcfDNA. The predictions were then compared with the actual diagnosis of BKPyVAN to assess the effectiveness of the predictions. These were used to develop algorithms to predict BKPyVAN using GcfDNA. Statistical analyses were performed using GraphPad Prism 8.0 and R version 4.0.3. All statistical tests adopted a 95% confidence interval (95% CI) and a two-tailed value of P< 0.05, indicating statistical significance.

## Results

### Patients and samples

**Figure 1:**
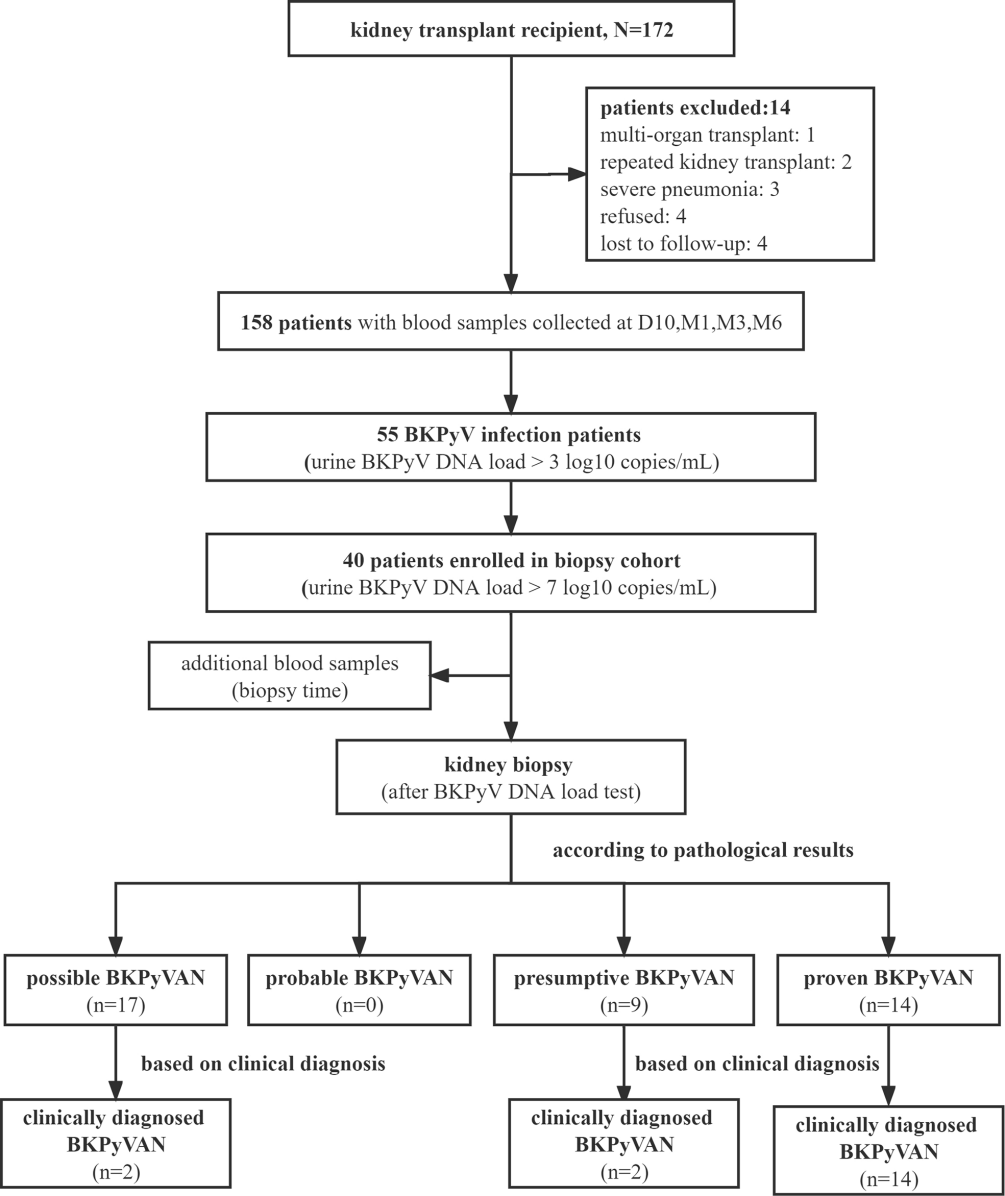
Consort diagram of patient flow through the study.

A flow diagram depicting the criteria used for patient inclusion is shown in **Figure 1**. A total number of 158 recipients with 672 plasma GcfDNA samples were included. The study data were collected until May 2022, with a median follow-up time of 325 (264, 408) days. The baseline information and indications between all patients and those with BKPyV infection were compared, and plasma GcfDNA (cp/mL) and GcfDNA (%) were not significantly different (**Table 2**).

**Table 2:** Patient characteristic.

Characteristic	All patients(n=158)	BKPyV infection patients(n=55)	P*
Baseline information			
Age (years)_doners, median (IQR)	49 (39, 54)	49 (44, 54)	0.973
Age (years)_recipients, mean ± SD	36 (28, 46)	38 (30, 45.5)	0.866
Serum creatinine levels before kidney transplant (μmol/L), median (IQR)	63.5 (43, 108.2)	65.7 (43.3, 90.35)	0.747
WIT (min), median (IQR)	6 (2, 14)	7 (2, 14.5)	0.922
CIT (min), median (IQR)	450 (180, 945)	400 (180, 740)	0.326
Donor type, n (%)			0.294
DBD	32 (15%)	6 (2.8%)	
DCD	63 (29.6%)	24 (11.3%)	
LD	63 (29.6%)	25 (11.7%)	
Indication for kidney transplant, n (%)			0.302
IgA nephropathy	12 (5.6%)	2 (0.9%)	
Nephrotic syndrome	84 (39.4%)	39 (18.3%)	
Chronic glomerulonephritis	2 (0.9%)	0 (0%)	
Diabetic nephropathy	1 (0.5%)	0 (0%)	
Others	11 (5.2%)	2 (0.9%)	
Not available	48 (22.5%)	12 (5.6%)	
GcfDNA(cp/mL), median (IQR)			
D10	0.64 (0.38, 1.00)	0.84 (0.47, 1.10)	0.142
M1	0.48 (0.29, 0.70)	0.48 (0.36, 0.75)	0.281
M3	0.45 (0.22, 0.67)	0.45 (0.20, 0.76)	0.606
M6	0.32 (0.17, 0.54)	0.28 (0.16, 0.55)	0.848
GcfDNA(%), median (IQR)			
D10	0.61 (0.40, 0.90)	0.7 0(0.40, 0.89)	0.883
M1	0.44 (0.30, 0.70)	0.50 (0.40, 0.78)	0.135
M3	0.40 (0.20, 0.60)	0.35 (0.18, 0.60)	0.475
M6	0.31 (0.20, 0.60)	0.30 (0.20, 0.70)	0.628

WIT, warm ischemia time; CIT, cold ischemia time; DBD, donor after brain death; DCD, donor after cardiac death; LD, living donor; BKPyV, BK polyomavirus; GcfDNA, graft-derived cell free DNA.

*Comparison between all patients and BKPyV infection patients.

According to the biopsy results, 17 were possible BKPyVAN, 9 were presumptive BKPyVAN, and 14 were proven BKPyVAN. No patient was diagnosed with probable BKPyVAN during the study period. Based on clinical judgment, in addition to 14 proven BKPyVAN, 2 out of 17 possible BKPyVAN and 2 out of 9 presumptive BKPyVAN were classified as clinically diagnosed BKPyVAN (n = 18). Serum creatinine levels before kidney transplant in the proven BKPyVAN group [85.8 (66.93, 124.2) µmol/L] were comparable to those in the presumptive BKPyVAN group [69.4 (52.3, 112.8) µmol/L, P = 0.53)] but greater than those in the possible BKPyVAN group [42.9 (41.2, 51.7) µmol/L, P< 0.001, P = 0.01]. The warm ischemia time (WIT) in the proven BKPyVAN group [11 (5.5, 14.75) min] was similar to that in the presumptive BKPyVAN group [8 (6, 12) min (P = 0.58)] but significantly higher than that in the possible BKPyVAN group [2 (2, 5) min (P = 0.006)]. The cold ischemia time (CIT) in the possible BKPyVAN group [230 (120, 480) min] was comparable to that in the presumptive BKPyVAN group [360 (240, 500) min (P = 0.33)] but was significantly lower than that in the proven BKPyVAN group [790 (440, 900) min (P = 0.007, P = 0.03)] (**Table 3**).

**Table 3:** Characteristic of possible BKPyVAN, presumptive BKPyVAN, and proven BKPyVAN patients.

Characteristic	Possible BKPyVAN(n=17)	Presumptive BKPyVAN(n=9)	Proven BKPyVAN(n=14)	P*
**Donor-associated parameters**				
Age(years), mean ± SD	49.29 ± 11.64	47.33 ± 4.95	49 ± 10.36	0.889
Sex, n (%)				0.009
Female	3 (7.5%)	3 (7.5%)	10 (25%)	
Male	14 (35%)	6 (15%)	4 (10%)	
BMI(kg/m^2^), mean ± SD	23.07 ± 3.16	22.49 ± 2.99	22.73 ± 3.28	0.899
Donor type, n (%)				< 0.001
DBD	0 (0%)	1 (2.5%)	5 (12.5%)	
DCD	3 (7.5%)	6 (15%)	6 (15%)	
LD	14 (35%)	2 (5%)	3 (7.5%)	
Serum creatinine levels before kidney transplant (μmol/L), median (IQR)	42.9 (41.2, 51.7)	69.4 (52.3, 112.8)	85.8 (66.93, 124.2)	0.001
WIT(min), median (IQR)	2 (2, 5)	8 (6, 12)	11 (5.5, 14.75)	0.015
CIT(min), median (IQR)	230 (120, 480)	360 (240, 500)	790 (440, 900)	0.012
**Recipient-associated parameters**				
Age(years), mean ± SD	32.47 ± 7.32	36.44 ± 9.11	42.64 ± 9.05	0.007
Sex, n (%)				0.078
Female	9 (22.5%)	4 (10%)	2 (5%)	
Male	8 (20%)	5 (12.5%)	12 (30%)	
BMI (kg/m^2^), median (IQR)	19.86 (18.37, 22.84)	22.32 (18.91, 22.64)	22.54 (20.69, 23.34)	0.276
**Matching parameters**				
HLA-MM, n (%)				< 0.001
0-3	16 (40%)	2 (5%)	4 (10.0%)	
4-6	1 (2.5%)	7 (17.5%)	10 (25.0%)	
DSA, n (%)				0.695
No	16 (40%)	8 (20%)	14 (35%)	
Yes	1 (2.5%)	1 (2.5%)	0 (0%)	
**Induction agent**				
Basiliximab, n (%)				0.533
No	4 (10%)	1 (2.5%)	1 (2.5%)	
Yes	13 (32.5%)	8 (20%)	13 (32.5%)	
r-ATG, n (%)				0.258
No	13 (32.5%)	9 (22.5%)	13 (32.5%)	
Yes	4 (10%)	0 (0%)	1 (2.5%)	
Rituximab, n (%)				1.000
No	16 (40%)	9 (22.5%)	13 (32.5%)	
Yes	1 (2.5%)	0 (0%)	1 (2.5%)	

BMI, body mass index; DBD, donor after brain death; DCD, donor after cardiac death; LD, living donor; WIT, warm ischemia time; CIT, cold ischemia time; HLA-MM, human leukocyte antigen mismatching; DSA, donor specific antibody; r-ATG, rabbit-antithymocyte globulin. *Comparison among possible BKPyVAN, presumptive BKPyVAN and proven BKPyVAN.

### Plasma GcfDNA (cp/mL) and GcfDNA (%) at biopsy time

**Figure 2:**
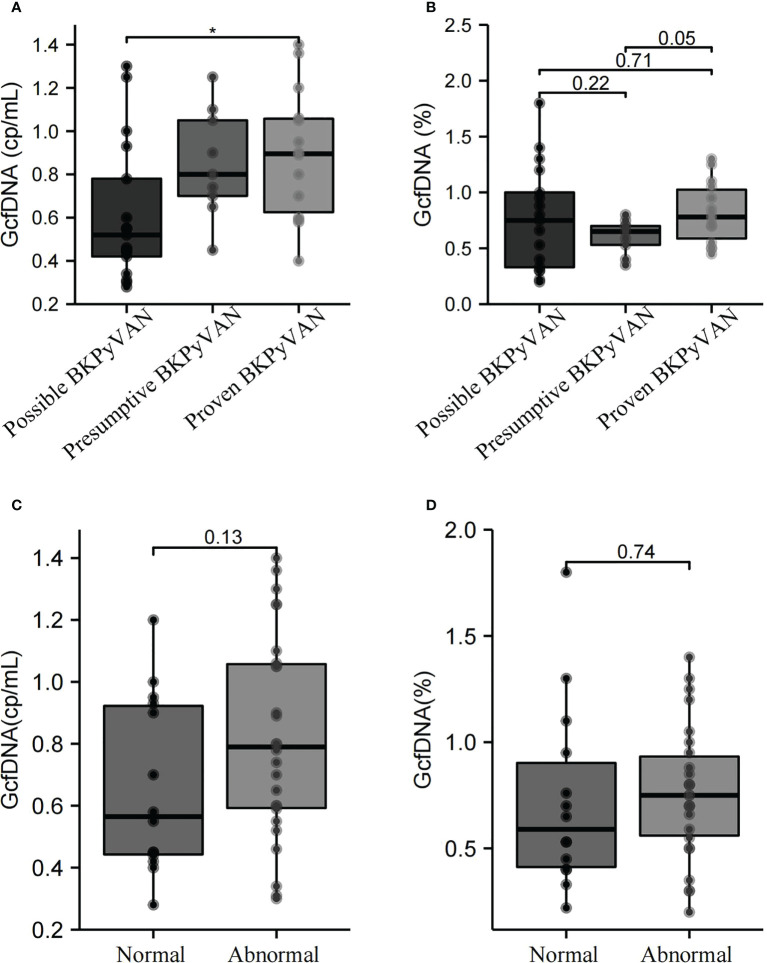
GcfDNA (cp/mL) and GcfDNA (%) in the possible BKPyVAN, presumptive BKPyVAN, and proven BKPyVAN groups were collected at biopsy time. **(A)** Boxplot with bold line represents the median GcfDNA (cp/mL) level in each subgroup; **(B)** Boxplot with bold line represents the median GcfDNA (%) level in each subgroup. GcfDNA (cp/mL) and GcfDNA (%) of between creatinine normal and abnormal groups. **(C)** Boxplot with bold line represents the median GcfDNA (cp/mL) level in normal creatinine group and abnormal creatinine group; **(D)** Boxplot with bold line represents the median GcfDNA (%) level in normal creatinine group and abnormal creatinine group. * P< 0.05.

At biopsy time, the median GcfDNA (cp/mL) in the proven BKPyVAN group [0.90 (0.63–1.06) cp/mL] was comparable to that in the presumptive BKPyVAN group [0.80 (0.70–1.05) cp/mL] but was significantly higher than that in the possible BKPyVAN group [0.52 (0.42–0.78) cp/mL (P = 0.037] (**Figure 2A**). However, no significant differences in median GcfDNA (%) were found between the possible BKPyVAN [0.75 (0.33–1.00)%], presumptive BKPyVAN [0.65 (0.53–0.70)%], and proven BKPyVAN [0.78 (0.59–1.03)%] groups (**Figure 2B**). Based on the patients’ creatinine levels, we divided them into a normal creatinine group (≤ 104 µmol/L) and an abnormal creatinine group (> 104 µmol/L). Our results revealed that there were no significant differences in plasma GcfDNA (cp/mL) (**Figure 2C**) and GcfDNA (%) (**Figure 2D**) between the normal and abnormal creatinine groups (P > 0.05).

### Diagnostic performance of plasma GcfDNA in BKPyVAN

**Figure 3:**
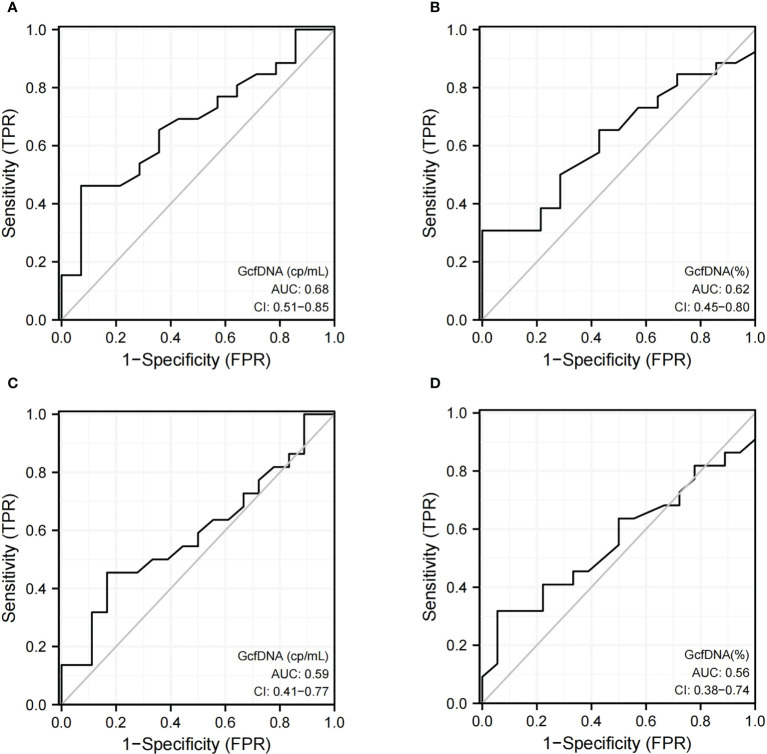
The discrimination capacity of GcfDNA (cp/mL) and GcfDNA (%) at biopsy time. Receiver operating curves of **(A)** GcfDNA (cp/mL) for distinguishing pathologically proven BKPyVAN(proven BKPyVAN) from pathologically unconfirmed BKPyVAN (possible BKPyVAN+presumptive BKPyVAN); **(B)** GcfDNA (%) for distinguishing pathologically proven BKPyVAN(proven BKPyVAN) from pathologically unconfirmed BKPyVAN (possible BKPyVAN+presumptive BKPyVAN); **(C)** GcfDNA (cp/mL) for distinguishing clinically diagnosed BKPyVAN from clinical excluded BKPyVAN; **(D)** GcfDNA (%) for distinguishing clinically diagnosed BKPyVAN from clinical excluded BKPyVAN.

The ROC curve showed the diagnostic performance of both GcfDNA (cp/mL) [AUC = 0.68, 95% CI: 0.51–0.85] and GcfDNA (%) [AUC = 0.62, 95% CI: 0.45–0.80] at biopsy time in distinguishing pathologically proven BKPyVAN from pathologically unconfirmed BKPyVAN. Moreover, the sensitivity and specificity of GcfDNA (cp/mL) were 92.9% and 46.2%, respectively, at a cutoff value of 0.57 cp/mL (**Figure 3A**). GcfDNA (%) had a cut-off value of 0.43%, with 92.9% sensitivity and 26.9% specificity (**Figure 3B**). The ROC curve showed the diagnostic performance of both GcfDNA (cp/mL) (**Figure 3C**) [AUC = 0.59, 95% CI: 0.41–0.77] and GcgDNA (%) (**Figure 3D**) [AUC = 0.56, 95% CI: 0.38–0.74] at biopsy time in distinguishing clinical diagnosed BKPyVAN from clinical excluded BKPyVAN.

It is widely recognized that GcfDNA elevation precedes clinical manifestations. We presume that this is the same for BKPyV infections. Therefore, we attempted to trace GcfDNA data before biopsy time. We focused on the latest programmed monitoring of GcfDNA (cp/mL) before biopsy time and baseline of GcfDNA (cp/mL) and found that 76.92% (30/39) of programmed monitoring values before biopsy were increased; plasma GcfDNA of programmed monitoring time and biopsy time of possible BKPyVAN, presumptive BKPyVAN, and proven BKPyVAN recipients are summarized in **Supplementary Table 1**. Therefore, we defined the individual change in GcfDNA (ΔGcfDNA) (cp/mL) to describe this increase. The formula used to calculate the change in GcfDNA is as follows:


ΔGcfDNA(cp/mL) = GcfDNA(cp/mL) of the latest programmed monitoring time before biopsy time – Baseline GcfDNA(cp/mL)

**Figure 4:**
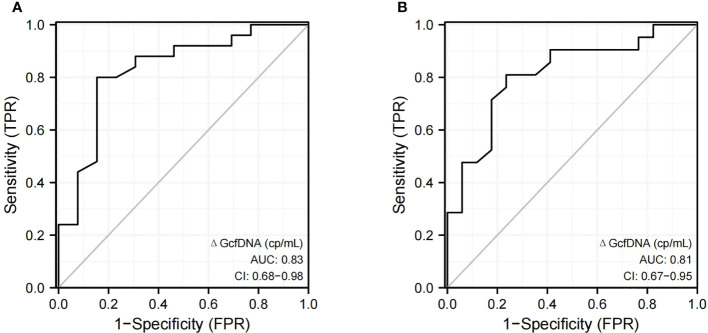
The discrimination capacity of ΔGcfDNA(cp/mL). Receiver operating curves of **(A)** ΔGcfDNA(cp/mL) for distinguishing pathologically proven BKPyVAN (proven BKPyVAN) from pathologically unconfirmed BKPyVAN (possible BKPyVAN+presumptive BKPyVAN); **(B)** ΔGcfDNA(cp/mL) for distinguishing clinically diagnosed BKPyVAN from clinical excluded BKPyVAN.

Two cases were excluded from the analysis: one patient was diagnosed with proven BKPyVAN on day 5 (baseline GcfDNA not available), and another patient was diagnosed with presumptive BKPyVAN on day 339 (long interval from programmed monitoring). The ROC curve showed that the diagnostic performance of ΔGcfDNA (cp/mL) had a sensitivity and specificity of 80% and 84.6%, respectively, when distinguishing pathologically proven BKPyVAN from pathologically unconfirmed BKPyVAN (AUC = 0.83) (**Figure 4A**), and a sensitivity and specificity of 81% and 76.5%, respectively, when distinguishing clinically diagnosed BKPyVAN from clinical excluded BKPyVAN (AUC = 0.81) (**Figure 4B**).

### Plasma ΔGcfDNA (cp/mL) in BKPyVAN and TCMR

**Figure 5:**
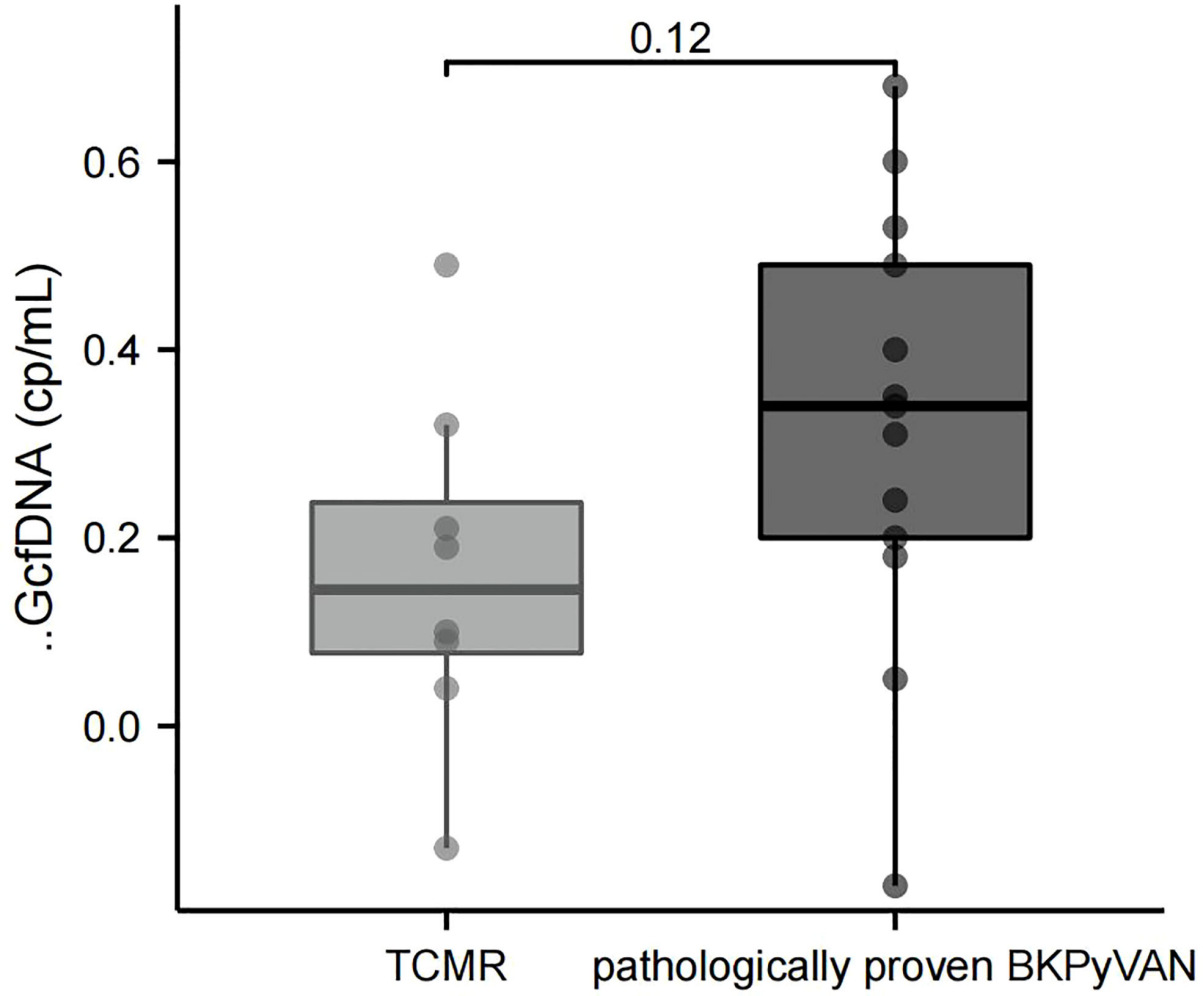
Comparison of ΔGcfDNA(cp/mL) between TCMR and pathologically proven BKPyVAN. BKPyVAN, BK polyomavirus-associated nephropathy; TCMR, T cell-mediated rejection.

It is known that the pathological changes of BKPyVAN and TCMR sometimes show many similarities, making the differential diagnosis very difficult. We wondered if ΔGcfDNA (cp/mL) could help distinguish them. Eight patients were diagnosed as biopsy-proven TCMR, We found programmed monitoring values before biopsy were also increased in the TCMR group, but plasma ΔGcfDNA (cp/mL) was not significantly different between TCMR [0.15 (0.08, 0.24) cp/mL] and pathologically proven BKPyVAN[0.34 (0.20, 0.49) cp/mL] (**Figure 5**).

### GcfDNA (cp/mL) is a predictor for pathologically proven BKPyVAN

In our previous studies, we found that early GcfDNA can predict eGFR values 90 days post-transplant (20). Furthermore, we intended to explore the efficacy of GcfDNA (cp/mL) of the first programmed monitoring (at D10) in predicting pathologically proven BKPyVAN. Consequently, we included relevant clinical variables (recipient age, donor age, serum creatinine level before transplant, WIT, CIT, HLA-MM, and creatinine level at D10) and GcfDNA (cp/mL) at D10 for LASSO regression analysis. LASSO regression was used to eliminate clinical variables that might overfit the model. We found that according to the coefficient distribution, recipients’ age, serum creatinine level before transplant, CIT, and GcfDNA (cp/mL) at D10 were included for model construction (**Supplementary Figure 1** and **Supplementary Table 2**). Our results revealed that the use of the LASSO regression model obtained high AUC values (0.89, 95% CI = 0.79–0.98) when predicting pathologically proven BKPyVAN, and the sensitivity and specificity were 0.93 and 0.69, respectively (**Supplementary Figure 2A**). The AUC of the LASSO regression model using clinical variables alone was 0.84 (**Supplementary Figure 2B**).

We further performed multivariate logistic regression to investigate whether GcfDNA alone had a predictive value. We include recipient age, donor age, serum creatinine level before transplant, WIT, CIT, HLA-MM, and creatinine level at D10 and GcfDNA at D10 in the model. And the multivariate logistic regression shows that GcfDNA at D10 (P = 0.007, OR = 13.04, 95% CI, 2.01–84.38) and HLA-MM (P = 0.021, OR = 7.92, 95% CI, 1.37–45.70) are independent predictors (**Supplementary Table 3**).

## Discussion

Plasma or urine GcfDNA levels can be used as non-invasive markers of kidney transplant injury; during ABMR, graft-damaged vascular endothelial cells directly release large amounts of GcfDNA into the circulation, followed by a simultaneous increase in plasma GcfDNA (cp/mL) and GcfDNA (%) (6, 7, 21). On the other hand, when BKPyV infection occurs in KTRs, changes in plasma GcfDNA (cp/mL) and GcfDNA (%) are less pronounced than those in ABMR, with only sporadic data reporting an abnormal rise (6, 10, 22–24). This is consistent with our findings, where we found that plasma GcfDNA (cp/mL) and GcfDNA (%) were not significantly elevated in 55 patients with BKPyV infection during the follow-up period. A likely reason for this is that BKPyV infection mainly causes damage to the renal tubular epithelium, and the GcfDNA released from the renal tubules does not enter the circulatory system until the development of BKPyVAN; therefore, the elevation of plasma GcfDNA is not significant. Many studies have suggested the use of urine GcfDNA for the diagnosis of BKPyVAN, and a study by Chen et al. has suggested that increased urine GcfDNA concentration is significantly correlated with BKPyVAN (12). However, the selection of urine GcfDNA has several limitations. First, cfDNA is easily decomposed and not easily preserved; the internal environment of urine is far less stable than that of blood, and its stable extraction is more challenging. Second, the volume of urine varies from patient to patient at different testing points in time, resulting in poor reproducibility of urine GcfDNA results. Finally, when combined with urinary tract infection, the total urine cfDNA is significantly elevated. Therefore, the GcfDNA test results are severely interfered with and do not accurately reflect the actual situation of kidney transplant damage due to BKPyV infection (25). Therefore, plasma GcfDNA rather than urine GcfDNA should be used to more accurately reflect the actual damage to the kidney transplant in BKPyVAN.

Our results were consistent with those reported by Chen (13), that plasma GcfDNA (cp/mL) was more diagnostic efficient than GcfDNA percentage (9, 26). With an AUC of 0.68 for pathologically proven BKPyVAN and an AUC of 0.62 for clinically diagnosed BKPyVAN, the ability of GcfDNA (cp/mL) at biopsy time to diagnose BKPyVAN was still relatively poor. As we know, subclinical injury is common among KTR and BKPyV infections and can cause graft damage before any clinical symptom, such as proteinuria or worsening creatinine (27). Therefore, it may not be appropriate to assess GcfDNA “for cause”. To further explore the use of plasma GcfDNA (cp/mL) in the diagnosis of BKPyVAN, we compared the changes in GcfDNA (cp/mL) values at the biopsy time and time of programmed monitoring. Interestingly, when compared to the baseline GcfDNA (cp/mL), 76.9% (30/39) of the programmed monitoring time GcfDNA (cp/mL) started to increase before biopsy time. The results support our hypothesis that GcfDNA (cp/mL) at the biopsy time is not a good representation of the onset of renal injury in BKPyVAN, and abnormal elevation of GcfDNA (cp/mL) caused by BKPyVAN should occur earlier. Moreover, to quantify the degree of this elevation, the baseline levels of GcfDNA should also be considered.

Considering patients’ programmed monitoring of GcfDNA on D10, M1, M3, and M6, we proposed a new algorithm for ΔGcfDNA (cp/mL), which is obtained by subtracting the baseline GcfDNA (cp/mL) from the measured GcfDNA(cp/mL) of the latest programmed monitoring time before biopsy time. The results showed that the diagnostic performance of ΔGcfDNA (cp/mL) has a better discriminatory ability for the diagnosis of BKPyVAN than GcfDNA (cp/mL) at the biopsy time, regardless of the diagnostic criteria of pathologically proven BKPyVAN or clinically diagnosed BKPyVAN. This result further suggests that BKPyVAN injury occurs before clinical manifestations and that programmed monitoring of plasma GcfDNA (cp/mL) may help identify kidney transplant injury earlier, thereby triggering earlier kidney biopsy and therapeutic intervention.

Shen et al. (28) found that urine GcfDNA can distinguish between BKPyVAN and type I TCMR. However, our results suggest that plasma ΔGcfDNA (cp/mL) cannot distinguish TCMR from pathologically proven BKPyVAN. This is also in line with the theory that GcfDNA is not specific to kidney allograft rejection which may be affected by different types of injury, the severity of the injury, and the detection time. In addition, the pathological changes of BKPyVAN and TCMR sometimes show many similarities, making the differential diagnosis very difficult. In other words, without information on virus load and dosage of an immunosuppressive agent, we question the practical value of GcfDNA in distinguishing between BK infection and TCMR. Moreover, a recent review suggests a threshold of 0.5% be considered for indicating TCMR (29). Our results showed that 29/40 patients with BKPyV infection have higher GcfDNA levels than 0.5% at biopsy time. This phenomenon again proves that GcfDNA cannot distinguish TCMR from pathologically proven BKPyVAN.

Early prediction of BKPyVAN can greatly improve its treatment outcomes. To our knowledge, this is the first study that combined clinical features with GcfDNA (cp/mL) to develop an algorithmic model for predicting BKPyVAN. Previous kidney transplant prediction models have been developed based on multiple sets of clinical data and algorithms, which could inform clinicians regarding graft rejection, graft acceptance, and patient survival (30). In the present study, using the LASSO regression model, which included clinical variables and GcfDNA biomarkers, we found that the clinical variables combined with GcfDNA (cp/mL) of the first programmed monitoring were better predictors of postoperative pathologically proven BKPyVAN than the clinical variables alone. Our results provide a new strategy for assessing and predicting various complications such as BKPyVAN after kidney transplantation. However, more data are required to validate this prediction model. Should the validation be successful, we expect to achieve real-time monitoring of individualized graft health after kidney transplant in clinical applications to improve the long-term survival of allogeneic kidney transplant recipients.

The main limitation of this study is that the number of patients recruited from a single hospital with a concurrent diagnosis of BKPyVAN was low, which requires the continued expansion of the cohort. Patients with multiorgan transplants, repeated kidney transplants, and severe pneumonia were not included in this study; therefore, these findings need to be extrapolated to these patients with caution.

In conclusion, this study found that plasma GcfDNA (cp/mL) and GcfDNA (%) in BKPyV-infected recipients did not differ significantly from all transplant patients in programmed monitoring. However, they showed significant elevation in proven BKPyVAN. Moreover, GcfDNA (cp/mL) has a low diagnostic efficacy for BKPyVAN at biopsy time. Our novel algorithms based on programmed monitoring, ΔGcfDNA (cp/mL), and the LASSO regression model show promising results in identifying and predicting BKPyVAN.

## Data availability statement

The raw data supporting the conclusions of this article will be made available by the authors, without undue reservation.

## Ethics statement

The studies involving human participants were reviewed and approved by Ethics Committee of the Sichuan Provincial People’s Hospital (No.2020405). The patients/participants provided their written informed consent to participate in this study.

## Author contributions

JW and YH conceived the study. RS and HY collected the data. JW and QR analyzed the data. JW wrote the manuscript. YH, HY, and QR reviewed and revised the manuscript. All authors contributed to the article and approved the submitted version.

## Funding

The study was supported by the “key R&D projects of Department of Science and Technology Sichuan Provincial” (no. 2022YFS0093) and “Youth Fund of Sichuan Provincial People’s Hospital” (no. 2022QN26).

## Acknowledgments

The authors thank Sichuan Provincial People’s Hospital, University of Electronic Science and Technology of China, for their support of this work and the reviewers for allowing us to make improvements to the manuscript.

## Conflict of interest

The authors declare that the research was conducted in the absence of any commercial or financial relationships that could be construed as a potential conflict of interest.

## Publisher’s note

All claims expressed in this article are solely those of the authors and do not necessarily represent those of their affiliated organizations, or those of the publisher, the editors and the reviewers. Any product that may be evaluated in this article, or claim that may be made by its manufacturer, is not guaranteed or endorsed by the publisher.

## References

[1] Sawinski D Goral S. BK virus infection: an update on diagnosis and treatment. Nephrology dialysis Transplant Off Publ Eur Dialysis Transplant Assoc - Eur Renal Assoc (2015) 30(2):209–17. doi: 10.1093/ndt/gfu023 24574543

[2] Yi SG Knight RJ Lunsford KE. BK virus as a mediator of graft dysfunction following kidney transplantation. Curr Opin Organ Transplant (2017) 22(4):320–7. doi: 10.1097/MOT.0000000000000429 28538243

[3] Snyder TM Khush KK Valantine HA Quake SR. Universal noninvasive detection of solid organ transplant rejection. Proc Natl Acad Sci United States America (2011) 108(15):6229–34. doi: 10.1073/pnas.1013924108 PMC3076856 21444804

[4] De Vlaminck I Valantine HA Snyder TM Strehl C Cohen G Luikart H. Circulating cell-free DNA enables noninvasive diagnosis of heart transplant rejection. Sci Trans Med (2014) 6(241):241ra277. doi: 10.1126/scitranslmed.3007803 PMC4326260 24944192

[5] Schütz E Fischer A Beck J Harden M Koch M Wuensch T. A noninvasive early rejection and graft damage marker in liver transplantation: A prospective, observational, multicenter cohort study. PloS Med (2017) 14(4):e1002286. doi: 10.1371/journal.pmed.1002286 28441386 PMC5404754

[6] Bloom RD Bromberg JS Poggio ED Bunnapradist S Langone AJ Sood P. Cell-free DNA and active rejection in kidney allografts. J Am Soc Nephrol JASN (2017) 28(7):2221–32. doi: 10.1681/ASN.2016091034 PMC5491290 28280140

[7] Sigdel TK Archila FA Constantin T Prins SA Liberto J Damm I. Optimizing detection of kidney transplant injury by assessment of donor-derived cell-free DNA *via* massively multiplex PCR. J Clin Med (2018) 8(1):19. doi: 10.3390/jcm8010019 PMC6352163 30583588

[8] Zhang H Zheng C Li X Fu Q Li J Su Q. Diagnostic performance of donor-derived plasma cell-free DNA fraction for antibody-mediated rejection in post renal transplant recipients: A prospective observational study. Front Immunol (2020) 11:342. doi: 10.3389/fimmu.2020.00342 32184785 PMC7058974

[9] Oellerich M Shipkova M Asendorf T Walson PD Schauerte V Mettenmeyer N. Absolute quantification of donor-derived cell-free DNA as a marker of rejection and graft injury in kidney transplantation: Results from a prospective observational study. Am J Transplant Off J Am Soc Transplant Am Soc Transplant Surgeons (2019) 19(11):3087–99. doi: 10.1111/ajt.15416 PMC6899936 31062511

[10] Whitlam JB Ling L Skene A Kanellis J Ierino FL Slater HR. Diagnostic application of kidney allograft-derived absolute cell-free DNA levels during transplant dysfunction. Am J Transplant Off J Am Soc Transplant Am Soc Transplant Surgeons (2019) 19(4):1037–49. doi: 10.1111/ajt.15142 30312536

[11] Xie WY Kim K Goussous N Drachenberg CB Scalea JR Weir MR. Causes of renal allograft injury in recipients with normal donor-derived cell-free DNA. Transplant direct (2021) 7(4):e679. doi: 10.1097/TXD.0000000000001135 33688578 PMC7935401

[12] Chen XT Qiu J Wu ZX Zhang H Chen T Yang SC. Using both plasma and urine donor-derived cell-free DNA to identify various renal allograft injuries. Clin Chem (2022) 68(6):814–25. doi: 10.1093/clinchem/hvac053 35587713

[13] Chen XT Chen WF Li J Deng RH Huang Y Yang SC. Urine donor-derived cell-free DNA helps discriminate BK polyomavirus-associated nephropathy in kidney transplant recipients with BK polyomavirus infection. Front Immunol (2020) 11:1763. doi: 10.3389/fimmu.2020.01763 32973745 PMC7466716

[14] Kant S Bromberg J Haas M Brennan D. Donor-derived cell-free DNA and the prediction of BK virus-associated nephropathy. Transplant direct (2020) 6(11):e622. doi: 10.1097/TXD.0000000000001061 33134498 PMC7587413

[15] Mayer KA Omic H Weseslindtner L Doberer K Reindl-Schwaighofer R Viard T. Levels of donor-derived cell-free DNA and chemokines in BK polyomavirus-associated nephropathy. Clin Transplant (2022) 27:e14785. doi: 10.1111/ctr.14785 PMC10078585 35894263

[16] Goussous N Xie W Dawany N Scalea JR Bartosic A Haririan A. Donor-derived cell-free DNA in infections in kidney transplant recipients: Case series. Transplant direct (2020) 6(7):e568. doi: 10.1097/TXD.0000000000001019 32766423 PMC7339327

[17] Hirsch HH Randhawa PS. BK polyomavirus in solid organ transplantation-guidelines from the American society of transplantation infectious diseases community of practice. Clin Transplant (2019) 33(9):e13528. doi: 10.1111/ctr.13528 30859620

[18] Haas M Loupy A Lefaucheur C Roufosse C Glotz D Seron D. The banff 2017 kidney meeting report: Revised diagnostic criteria for chronic active T cell-mediated rejection, antibody-mediated rejection, and prospects for integrative endpoints for next-generation clinical trials. Am J Transplant Off J Am Soc Transplant Am Soc Transplant Surgeons (2018) 18(2):293–307. doi: 10.1111/ajt.14625 PMC5817248 29243394

[19] Hirsch HH Randhawa P. BK polyomavirus in solid organ transplantation. Am J Transplant Off J Am Soc Transplant Am Soc Transplant Surgeons (2013) 13 Suppl 4:179–88. doi: 10.1111/ajt.12110 23465010

[20] Di W Ran Q Yang H Lu J Hou Y Wang X. Use of graft-derived cell-free DNA as a novel biomarker to predict allograft function after kidney transplantation. Int J Urol Off J Japanese Urological Assoc (2021) 28(10):1019–25. doi: 10.1111/iju.14638 34229363

[21] Huang E Sethi S Peng A Najjar R Mirocha J Haas M. Early clinical experience using donor-derived cell-free DNA to detect rejection in kidney transplant recipients. Am J Transplant Off J Am Soc Transplant Am Soc Transplant Surgeons (2019) 19(6):1663–70. doi: 10.1111/ajt.15289 30725531

[22] Shen J Zhou Y Chen Y Li X Lei W Ge J. Dynamics of early post-operative plasma ddcfDNA levels in kidney transplantation: a single-center pilot study. Transplant Int Off J Eur Soc Organ Transplant (2019) 32(2):184–92. doi: 10.1111/tri.13341 30198148

[23] Bu L Gupta G Pai A Anand S Stites E Moinuddin I. Clinical outcomes from the assessing donor-derived cell-free DNA monitoring insights of kidney allografts with longitudinal surveillance (ADMIRAL) study. Kidney Int (2022) 101(4):793–803. doi: 10.1016/j.kint.2021.11.034 34953773

[24] Gielis EM Ledeganck KJ Dendooven A Meysman P Beirnaert C Laukens K. The use of plasma donor-derived, cell-free DNA to monitor acute rejection after kidney transplantation. Nephrology dialysis Transplant Off Publ Eur Dialysis Transplant Assoc - Eur Renal Assoc (2020) 35(4):714–21. doi: 10.1093/ndt/gfz091 31106364

[25] Burnham P Dadhania D Heyang M Chen F Westblade LF Suthanthiran M. Urinary cell-free DNA is a versatile analyte for monitoring infections of the urinary tract. Nat Commun (2018) 9(1):2412. doi: 10.1038/s41467-018-04745-0 29925834 PMC6010457

[26] Sun K Jiang P Chan KC Wong J Cheng YK Liang RH. Plasma DNA tissue mapping by genome-wide methylation sequencing for noninvasive prenatal, cancer, and transplantation assessments. Proc Natl Acad Sci United States America (2015) 112(40):E5503–5512. doi: 10.1073/pnas.1508736112 PMC4603482 26392541

[27] Lubetzky ML Salinas T Schwartz JE Suthanthiran M. Urinary cell mRNA profiles predictive of human kidney allograft status. Clin J Am Soc Nephrol CJASN (2021) 16(10):1565–77. doi: 10.2215/CJN.14010820 PMC8499006 33906907

[28] Shen J Guo L Lei W Liu S Yan P Liu H. Urinary donor-derived cell-free DNA as a non-invasive biomarker for BK polyomavirus-associated nephropathy. J Zhejiang Univ Sci B (2021) 22(11):917–28. doi: 10.1631/jzus.B2100131 PMC8593525 34783222

[29] Kant S Brennan DC. Donor derived cell free DNA in kidney transplantation: The circa 2020-2021 update. Transplant Int Off J Eur Soc Organ Transplant (2022) 35:10448. doi: 10.3389/ti.2022.10448 PMC9198901 35721467

[30] Tapak L Hamidi O Amini P Poorolajal J. Prediction of kidney graft rejection using artificial neural network. Healthcare Inf Res (2017) 23(4):277–84. doi: 10.4258/hir.2017.23.4.277 PMC5688027 29181237

