# Supplementary material for: Detection of BK polyomavirus-associated nephropathy using plasma graft-derived cell-free DNA: Development of a novel algorithm from programmed monitoring
Source: Front Immunol. 2022 Oct 6;13:1006970. doi: 10.3389/fimmu.2022.1006970 (PMC9582120; doi:10.3389/fimmu.2022.1006970)
Supplement: Supplementary file 1 [file DataSheet_1.docx]

Supplementary Material

# Supplementary Figures and Tables

## Supplementary Figures 1

**
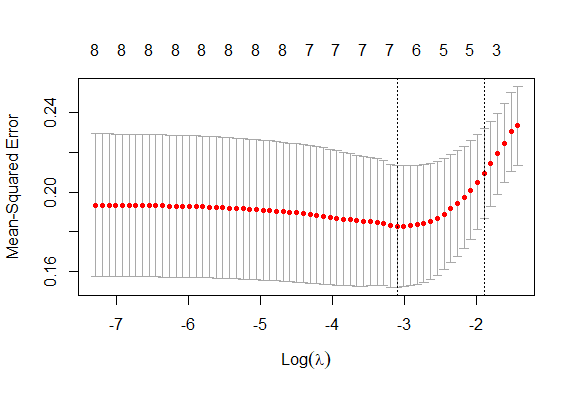
**

**Supplementary Figure 1.** LASSO coefficient screening.

## Supplementary Figures 2

**
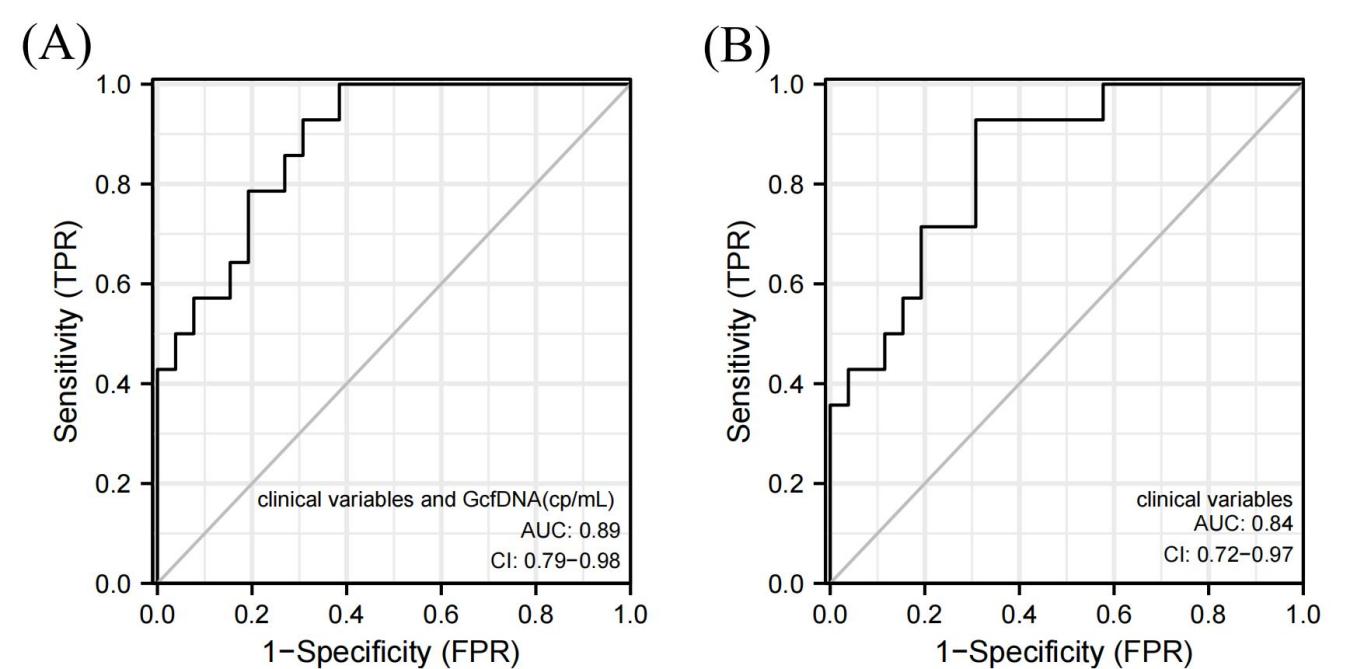
**

**Supplementary Figure 2.** The ROC curve of predicting the pathological BKPyVAN (A) construct the model with clinical variables and early GcfDNA(cp/mL) ; (B) construct the model with clinical variables alone.

## Supplementary Tables

**Supplementary Table 1.** Programmed monitor time and biopsy time of GcfDNA (cp/mL) in the possible BKPyVAN，presumptive BKPyVAN，and proven BKPyVAN recipients

| **Characteristics** | **D10** | **M1** | **M3** | **M6** | **biopsy time** | **days** |
| --- | --- | --- | --- | --- | --- | --- |
| **possible BKPyVAN** | | | | | |  |
| patient 1 | 0.44 | 0.64 | 0.14 | 0.20 | 0.78 | 81 |
| patient 2 | 0.46 | 0.97 | 0.42 | 0.30 | 0.93 | 40 |
| patient 3 | 1.00 | 0.28 | 0.80 | 0.60 | 0.45 | 195 |
| patient 4 | 0.38 | 0.85 | 0.60 | 0.25 | 0.60 | 213 |
| patient 5 | 0.50 | 0.65 | 0.20 | 0.14 | 0.46 | 44 |
| patient 6 | 1.30 | 0.48 | 0.55 | 0.80 | 0.42 | 95 |
| patient 7 | 0.90 | 0.90 | 0.11 | 1.00 | 0.44 | 102 |
| patient 8 | 0.21 | 0.31 | 0.38 | 0.50 | 0.34 | 205 |
| patient 9 | 0.29 | 0.35 | 0.56 | 0.42 | 0.55 | 53 |
| patient 10 | 0.48 | 0.60 | 1.90 | 0.80 | 0.28 | 81 |
| patient 11 | 0.60 | 0.68 | 0.44 | 0.32 | 0.52 | 55 |
| patient 12 | 0.33 | 0.47 | 0.40 | 0.10 | 1.25 | 87 |
| patient 13 | 0.40 | 0.40 | 0.83 | 0.14 | 1.00 | 98 |
| patient 14 | 1.20 | 0.59 | 0.90 | 0.50 | 0.55 | 191 |
| patient 15 | 1.20 | 0.66 | 0.88 | 0.44 | 0.31 | 126 |
| patient 16 | 0.16 | 0.95 | 1.05 | 0.33 | 1.30 | 91 |
| patient 17 | 0.90 | 0.54 | 0.11 | 0.11 | 0.30 | 134 |
| **Presumptive BKPyVAN** | | | | | |  |
| patient 18 | 0.56 | 0.68 | 0.20 | 0.17 | 0.80 | 126 |
| patient 19 | 0.90 | 0.80 | 0.80 | 0.33 | 1.05 | 155 |
| patient 20 | 0.79 | 0.65 | 0.60 | 0.24 | 0.70 | 122 |
| patient 21 | 0.81 | 0.40 | 0.45 | 0.55 | 0.90 | 177 |
| patient 22 | 0.78 | 0.58 | 0.60 | 0.24 | 0.45 | 156 |
| patient 23 | 0.84 | 0.45 | 0.77 | 0.10 | 0.74 | 113 |
| patient 24 | 1.30 | 0.17 | 0.53 | 0.38 | 1.25 | 256 |
| patient 25 | 0.56 | 0.82 | 0.76 | 0.80 | 0.65 | 181 |
| patient 26 | 0.49 | 0.29 | 0.33 | 1.3 | 1.1 | 339 |
| **Proven BKPyVAN** | | | | | |  |
| patient 27 | 0.74 | 1.05 | 0.77 | 0.66 | 1.36 | 73 |
| patient 28 | 1.30 | 1.13 | 0.21 | 0.15 | 0.89 | 33 |
| patient 29 | 1.10 | 0.50 | 0.90 | 0.14 | 1.06 | 103 |
| patient 30 | 1.40 | 1.58 | 0.10 | 0.08 | 1.05 | 74 |
| patient 31 | 0.85 | 0.90 | 0.20 | 0.15 | 0.59 | 86 |
| patient 32 | 0.88 | 0.62 | 1.30 | 0.16 | 0.80 | 147 |
| patient 33 | 1.00 | 1.35 | 0.58 | 0.42 | 0.70 | 48 |
| patient 34 | 1.10 | 1.34 | 0.63 | 0.55 | 0.90 | 77 |
| patient 35 | 0.95 | 0.32 | 0.85 | 0.27 | 0.60 | 122 |
| patient 36 | 0.90 | 0.46 | 0.95 | 0.60 | 0.58 | 96 |
| patient 37 | 1.15 | 1.49 | 0.23 | 0.15 | 1.20 | 40 |
| patient 38 | 1.20 | 0.40 | 0.60 | 0.82 | 0.40 | 96 |
| patient 39 | 1.05 | 0.80 | 1.40 | 0.28 | 1.40 | 116 |
| patient 40 | 0.85 | 0.4 | 0.08 | 0.1 | 0.95 | 5 |

BKPyVAN, BK polyomavirus-associated nephropathy; D10, on the tenth day; M1, on the first month; M3, on the third month; M6, on the sixth month.

**Supplementary Table 2.**Coefficients of each variable by LASSO regression

| **feature** | **coef** |
| --- | --- |
| (intercept) | -4.613558e-03 |
| Recipients’ age | 3.967646e-03 |
| Doners’ age | 0.000000000 |
| Serum creatinine levels before kidney transplant | 9.603682e-05 |
| WIT | 0.000000000 |
| CIT | 5.611208e-05 |
| HLA-MM | 0.000000000 |
| Creatinine at D10 | 0.000000000 |
| GcfDNA(cp/mL) at D10 | 2.131635e-01 |

WIT: warm ischemia time; CIT: cold ischemia time; HLA-MM, human leukocyte antigen mismatch; GcfDNA, Graft-derived cell-free DN; D10, on the tenth day.

**Supplementary Table 3. Logistic regression in multivariate analysis**

| **Risk factor** | **Odds ratio (95% CI)** | ***P* value** |
| --- | --- | --- |
| GcfDNA(cp/mL) at D10 | 13.04 (2.01-84.38) | 0.007 |
| HLA-MM | 7.92 (1.37-45.70) | 0.021 |

GcfDNA, Graft-derived cell-free DN; D10, on the tenth day; HLA-MM, human leukocyte antigen mismatch.
